# Transforming Lives: A Qualitative Study Seeking Insights Into Reconstructive Surgery for Leprosy Patients in Western Maharashtra

**DOI:** 10.7759/cureus.68416

**Published:** 2024-09-01

**Authors:** Kajal Shrivastava, Suman Ray, Hetal Rathod, Abhijeet L Wahegaonkar, Sudhir L Jadhav, Anil Mahajan, Johnson S, Prerna Verma, Deepu Palal, Shreya Sengupta

**Affiliations:** 1 Community Medicine, Dr. D. Y. Patil Medical College, Hospital and Research Centre, Dr. D. Y. Patil Vidyapeeth, Pune, IND; 2 Orthopaedics, Sahyadri Super Speciality Hospital, Pune, IND

**Keywords:** societal stigma, physical deformities, rehabilitation services, leprosy complications, plastic and reconstructive surgery, national leprosy eradication programme

## Abstract

Introduction

Leprosy remains a significant health issue, especially in areas where diagnosis and treatment face numerous barriers, leading to preventable deformities and disabilities. This study aims to explore the obstacles to reconstructive surgery for leprosy patients, from both patient and healthcare provider perspectives. By conducting a qualitative analysis, the study seeks to assess the impact of reconstructive surgery on patients' quality of life, encompassing their physical, psychological, emotional, and social well-being.

Methods

This qualitative study was conducted from April to May 2024. One focus group discussion (FGD) for 12 participants, along with two in-depth interviews, was conducted for the patients at a leprosy rehabilitation center in western Maharashtra who have completed leprosy treatment and have undergone reconstructive surgeries for their disability. One in-depth interview was conducted with the key informant (a healthcare provider who is a surgeon who performs reconstructive surgeries for leprosy patients). Participants were selected through purposive sampling until information saturation was achieved. Interviews were conducted in local languages and analyzed using thematic analysis to identify key barriers and themes.

Results

A qualitative analysis of feedback from leprosy patients who underwent reconstructive surgery (RCS) highlights the importance of family support and the transformative impact of surgery on functionality and psychological well-being. Stigmatization and fear often delayed treatment-seeking behavior, but government incentives alleviated economic burdens, and participants expressed readiness to recommend RCS to others. Surgeons emphasize the variety of surgeries performed, eligibility criteria, recovery period, and success rate of 85-90%, noting the importance of financial accessibility and a multidisciplinary approach. Suggestions for improvement include infrastructure enhancement, adequate funding, and active case detection by the National Leprosy Eradication Programme (NLEP).

Conclusion

The findings highlight the complex interplay of factors contributing to delays in reconstructive surgery for leprosy patients in India. Addressing these barriers requires multifaceted interventions, including increasing public awareness, improving healthcare infrastructure, and enhancing support systems for patients. Policy development should focus on these areas to reduce disparities and improve the outcomes of reconstructive surgery in resource-limited settings.

## Introduction

Leprosy, also known as Hansen’s disease, is a chronic, infectious tropical ailment classified as a neglected tropical disease (NTD). It stems from an infection by the bacterium *Mycobacterium leprae* and manifests in the skin, peripheral nerves, nasal mucosa, and eyes [1,2].

Leprosy remains prevalent in over 120 countries, with an annual report of over 200,000 new cases. Globally, the elimination of leprosy as a public health concern, defined as a prevalence of less than 1 per 10,000 people, was accomplished in 2000 according to World Health Assembly resolution 44.9 and in most nations by 2010. The decline in new case numbers has been gradual, both worldwide and across WHO regions. By 2019, India, Indonesia, and Brazil reported more than 10,000 new cases each, while 13 countries (The Democratic Republic of the Congo, Bangladesh, Madagascar, Mozambique, Ethiopia, Myanmar, Nigeria, Nepal, The Philippines, Sri Lanka, Somalia, South Sudan, and the United Republic of Tanzania) reported between 1,000 and 10,000 new cases. Forty-five countries reported no cases, and 99 reported fewer than 1,000 new cases [1].

India has had considerable success in its long fight against leprosy. This has included breakthroughs in leprology as a scientific field as well as successes in leprosy management at the levels of public health and clinical practice. The first two decades following the debut of multi-drug therapy (MDT) in India in 1983 were marked by extraordinary success. The path has been turbulent yet fulfilling, as the cases have fallen from 57.6 per 10,000 population in 1981 to less than one case per 10,000 in 2005 [3,4]. The current prevalence rate of leprosy is 0.4 per 10,000 people in the country, with an active case detection rate of 4.56 per 10,000 people [5,6]. It has been declared to have been eliminated as a major public health concern. However, data released recently indicated that 53.6% of new cases reported worldwide last year originated from India [7].

In the years 2022-2023, Maharashtra reported approximately 18,000 cases of leprosy, following the detection of nearly 17,000 cases prior to that period, as confirmed by state health department officials. Additionally, a comprehensive house-to-house survey conducted from November 20 to December 6 last year revealed over 6,500 cases [8]. In 2023, Maharashtra witnessed a 7% increase in leprosy cases among children [9]. The endemic regions of Maharashtra are within the Nagpur Circle (comprising Nagpur, Chandrapur, Gadchiroli, and Gondia), the Mumbai Circle (including Thane, Palghar, and Raigad), and the Marathwada and Pune regions [10].

Leprosy, unlike many other contagious diseases, evokes not only fear of transmission but also a sense of horror and revulsion, leading to avoidance and social exclusion. The stigma associated with leprosy can be described as the erosion or distortion of the patient's true identity as an individual. Early diagnosis of leprosy can prevent visible deformities or functional impairments. However, the prevailing perception of leprosy as a lifelong debilitating condition persists, reinforcing the perceived necessity of concealing the diagnosis until intervention becomes less effective [11].

Leprosy imposes a multifaceted burden on individuals' quality of life. Physically, it ravages the skin and peripheral nerves, manifesting as lesions, numbness, and muscle weakness, often leading to debilitating injuries and disfigurement if left untreated. Psychologically, leprosy perpetuates stigma and discrimination, fostering fear and misconceptions that ostracize sufferers from their communities and fuel mental health struggles such as anxiety, depression, and low self-esteem. Socially, it disrupts relationships, fostering strained connections with family and friends and perpetuating economic instability due to reduced employability, thereby exacerbating poverty and limiting access to essential resources and healthcare. Access to adequate medical care is often hindered by regional constraints and stigma, resulting in delayed diagnosis and suboptimal treatment, further deteriorating individuals' well-being.

Despite these challenges, addressing stigma and discrimination while providing comprehensive medical care, rehabilitation, and psychosocial support remains pivotal in enhancing the quality of life for those affected by leprosy. Activities that empower patients and the community via information, education, and communication (IEC) are necessary because they have a profoundly healing and de-stigmatizing effect on both the patients and the community at large, which will help in active case detection and prompt treatment [12].

Reconstructive surgery in leprosy 

Leprosy often affects the nerves, leading to deformities in the hands, feet, or eyes, which result in disabilities for those afflicted. Individuals who have been cured of leprosy but are left with these deformities may need reconstructive surgery (RCS) to enhance their functional abilities. Successful surgical outcomes require pre- and post-operative physiotherapy, making it a crucial component of the RCS process. These deformities contribute to ongoing stigma and discrimination, making early correction a priority.

Reconstructive surgeries for leprosy patients include various procedures for different parts of the body. For the hand, surgeries encompass claw correction, opponenplasty for the thumb, wrist drop correction, stabilization procedures such as arthrodesis, and tissue reconstruction procedures like contracture release and flap cover. For the foot, the surgeries include foot drop correction, claw toe correction, soft tissue reconstruction of the sole, and stabilization procedures such as arthrodesis. Eye-related surgeries focus on the correction of lagophthalmos. For the nose, reconstructive procedures address the reconstruction of a collapsed nose. All of these aim to restore both function and form to the greatest extent possible, preventing further disability and playing a vital role in rehabilitation. This surgery helps leprosy-affected individuals regain their status in the community, thereby reducing the stigma associated with the disease. NGO institutions that conduct reconstructive surgery often report that leprosy-affected individuals with disabilities, who are typically poor, are reluctant to undergo surgery despite it being provided free of charge. This reluctance is due to the required long hospital stays and the economic burden on family members who need to accompany and stay with the patients.

To address these challenges, an incentive of Rs 8,000/- which is now increased to 12,000/- is provided to leprosy-affected individuals. This incentive is available to all patients from below poverty line (BPL) families, regardless of whether they are operated on in a Government hospital or NGO institution. Rehabilitation and postoperative care are crucial for patients undergoing reconstructive surgery for leprosy. Physiotherapy is essential for regaining strength and function in the affected limbs, while occupational therapy helps patients adapt to any physical limitations and improve their ability to perform daily activities. Additionally, psychological support is vital to address the mental and emotional impact of the disease and its physical consequences, ensuring a holistic approach to patient recovery and well-being. Therefore, it is crucial to regularly review the operated cases for at least six months after the surgery [13].

Given the clinical significance of delayed diagnosis and the limited understanding of region-specific barriers to early detection and treatment of leprosy, which ultimately leads to deformities and disability, there is an urgent need for comprehensive research in this area. This study aims to examine the obstacles perceived by both patients and healthcare providers concerning reconstructive surgery in leprosy patients. Conducting a qualitative study will allow us to assess the impact of reconstructive surgery on patients' quality of life, addressing both the physical and psychological, emotional, and social dimensions of their experiences.

## Materials and methods

Study design and setting

This was a qualitative study that was conducted from April 2024 to May 2024 at a leprosy rehabilitation centre in western Maharashtra, India. 

Ethical considerations

Before the study began, ethical clearance was obtained from the Institutional Review Board of Dr. D. Y. Patil Medical College, Hospital and Research Centre, Pune, with approval number IESC/PGS/2022/204. Participants were fully informed about the study's objectives and data collection methods to ensure transparency, and their confidentiality was maintained through a system of codes and numbers. Informed written consent was obtained from each patient or their caregiver before the study commenced.

Data collection

One focus group discussion (FGD) for 12 participants, along with two in-depth interviews, was conducted for the patients at a leprosy rehabilitation centre in western Maharashtra who have completed leprosy treatment and have undergone reconstructive surgeries for their disability. Convenience sampling was used to select the participants. One in-depth interview was conducted for a key informant (a healthcare provider who is a surgeon who performs reconstructive surgeries for leprosy patients) using an interview guide at a convenient place and time in the vernacular language or English or Hindi as applicable. Consent was obtained for audio/video recording.

Preparatory phase

An interview guide was prepared for the FGD and the in-depth interviews. A team was prepared for conducting the FGD and interviews, which included a moderator (who remained the same for the FGD and interviews for the patients), translator, assistant, note-taker, and audio-visual recorder.

Recruitment of participants

Key informants were selected from the rehabilitation center in Pune. For the FGD and in-depth interviews, a diverse group of participants was carefully planned, comprising patients who had undergone various types of reconstructive surgeries. To facilitate effective communication and interaction between the participants and the interviewer, a semi-circular seating arrangement was adopted. Only participants aged 18 years and above who had undergone reconstructive surgery at least six months prior were included in the study to ensure that they had sufficient time to recover and reflect on their experiences.

Interview process

Greetings and icebreakers were part of a formal introduction, followed by the conversation itself. The participants were informed that the data collected would be anonymized and kept confidential, and the identity of the participants would not be shared with the higher authorities. Each person had an equal chance to respond to the questions asked. The study participants were questioned until data saturation was obtained. The study participants were given the chance to ask questions, and their concerns were answered. The note-taker provided notes that were verbatim accurate.

Data analysis

Data from patients and the healthcare provider (surgeon) were explored by thematic analysis. The process of data analysis began with the transcription of audio and video recordings, which were transcribed verbatims. Researchers then familiarized themselves with the data by reading the transcripts multiple times. Initial codes were systematically generated across the entire data set, followed by the development of potential themes by collating codes and gathering all relevant data. These themes were reviewed and refined to ensure they accurately reflected the data, and clear definitions and names were assigned to each theme. The final step involved a detailed write-up of the themes, supported by quotes from the participants, to provide a comprehensive understanding of the experiences and perspectives of the patients and the healthcare provider.

## Results

Leprosy patients' qualitative feedback through a focus group discussion

**Figure 1:**
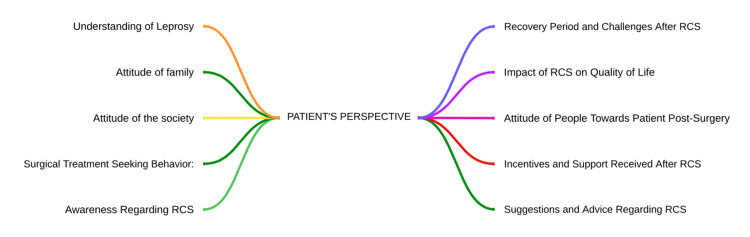
Themes generated from the focused group discussion (FGD)

Qualitative feedback from the leprosy patients who underwent reconstructive surgery (RCS) was collected through a focus group discussion. One focused group discussion was done at the rehabilitation centre, where 12 patients who underwent reconstructive surgery were interviewed. The responses provided offer a comprehensive insight into the patient's perspective regarding leprosy diagnosis, patient surgical treatment-seeking behavior, access to information about reconstructive surgery (RCS), recovery period post-surgery, the impact of RCS on life, changes in the attitude of people around post-surgery, incentives received after RCS, and advice for someone considering RCS. These highlight the complex interplay of emotions, experiences, and practical considerations shaping the patient's journey with leprosy and reconstructive surgery. Following this, themes were generated, which are depicted in Figure 1, and the verbatims were quoted as provided by the interviewees, which are provided below. 

Understanding of Leprosy

During the focus group discussion, participants were asked about their understanding of leprosy. Most expressed a deep conviction that leprosy is a hardship that no one should have to endure, underscoring the severe impact of the disease. The deformities resulting from leprosy were seen as indicators of a serious illness, further highlighting its gravity. The sadness caused by job loss emphasized the significant social and economic challenges associated with the disease. Even with family support, patients often still experience profound distress, which drives them to seek the specialized care and mental health support provided by rehabilitation centers.

Patient 1: *“Mujhe yeh lagta hai, ki yeh khusth rog hai, woh kisiko nai hona chahiye”* (I feel no one should ever have leprosy disease).

Patient 4: “*Mere ko yeh samaj main aata hai, yeh kushth rog saans lene se felta hai*” (I have come to understand that leprosy spreads by breathing in the same room).

Patient 5: *"Kushthaogamule mazi nokari gamavlyamule mi khup dukhi ahe*”(I feel sad because losing my job due to leprosy has been incredibly difficult for me).

Patient 8: *“Maine yeh maan lia hai ki yeh vikrti kusth rog ke wajah se”* (I have accepted that my deformities is due to leprosy).

Patient 12: *"Kutumbachya samarthan asun suddha, mala vait watat hota ani tyamule me kondhwa madhe janayacha nirnay ghetla"* (Despite my family's support, I still felt bad and decided to go to Kondhwa).

Attitude of the Family 

While some families struggle with stigma, fear, and misinformation, others demonstrate unwavering support and acceptance of leprosy patients. Family engagement is crucial for leprosy patients within communities, as it plays a significant role in mitigating the clinical, psychological, social, and behavioral impacts of the disease and determines the quality of life. The participants were asked about the attitude of their family towards them.

Patient 2: *“Mera parivaar mera sabase adhik samarthan nahi karata tha. Rishtedaar bahut gusse mein the aur baat itanee badh gaee ki mujhe ghar se baahar nikaal diya gaya”* My family didn't support me when I needed them the most. (Relatives were angry, and it got so bad that I was thrown out of the house).

Patient 5: *“Majhe palak samarthaka hote, aṇi tyanni majhe changale sangopana kele. Khara tara, majhya vaḍilanni ayurveda upachar suruvata kele hote"* (My parents were supportive and treated me well. In fact, my father initiated Ayurveda treatment).

Patient 6: *“Majham vivahavar prabhav jhala. majhi patni mala sodun geli”* (My marriage was affected and my wife left me).

Attitude of People of the Society

Lack of awareness, entrenched beliefs, fear, and shame have contributed to the stigmatization of leprosy. This stigma leads to unjust treatment of individuals affected by the disease, causing emotional distress and fostering discriminatory actions. Participants were asked about the perception society had towards them.

Patient 1: *"Main rameshawarama gaya tha. Udhar mere vikriti ko dekh ke mujhe chai bhi nai dia ek dukan main"* (I went to Rameshwaram; I wasn’t served tea at a stall for my deformity).

Patient 3: *“Shejarchyani anandavana jayacha salla dila”* (Neighbors advised me to go to anandvan).

Patient 5: *“Naukarituna bahera khadlyanantar, majhya dona sahakarimitrani mala okaran kade jayacha salla dila” (*After being removed from my job, two of my colleagues advised me to see a doctor).

Surgical Treatment Seeking Behaviour 

Leprosy patients often delay seeking treatment due to stigma, misconceptions, and fear of diagnosis. This delay can lead to worsening symptoms and disabilities. Once that sets in, it affects their quality of life and income, and that leads them to seek surgical intervention in the form of reconstructive surgeries (RCS). Here, participants were asked about their surgical treatment-seeking behavior.

Patient 7: *“Mala kaam karṇyacha ashakya asalyane, oparesan karaṇe avashyak hote.”* (Getting operated was necessary as I was not able to work at all.)

Patient 9: *“Anna khaṇyasaṭhihi mala madatici avasyakta hoti, tyamule oparesan avasyak hote.”* (Operation was necessary as I needed help to even have food.)

Patient 10: *“Mala majhya dainandin kamansaṭhi madatici avasyakta hoti. Mi svataḥ ann khaṇyasaṭhi kiṁva majhya kapaḍyanna ghalanyasathi sakṣham navhato*” (I needed help for my daily chores. I was not able to eat or wear my clothes on my own).

Patient 11: *“Hya rogacha ani tyacha asamaika mule mala sheti sodana anivarya hota”* (I had to leave farming because of this disease and its deformities).

Awareness Regarding RCS 

Reconstructive surgery is an important part of the National Leprosy Eradication Program. Health campaigns and education programs strive to enhance awareness and encourage patients. Despite these efforts, patients requiring correction for their deformities have not been very responsive, and there appears to be a general reluctance towards hospitalization and surgery. Here, the participants were asked about their awareness regarding RCS and how they got information for doing it.

Patient 2: *"Majhya doctoranni mala kushtarog rugnalayat janyachi mahiti dili, jithe majhi shastrakriya hou shakte."* (Information was given by my doctor to go to leprosy hospital where my surgery can happen.)

Patient 6:* "Madhye aalyanantar, doctoranni mala shastrakriye, babat sangitla ani karnyacha salah dila."* (After coming to the rehab centre, doctor mentioned and counselled me about getting operated.)

Patient 9: *"Anandwan madhe lokanni mala tyachi mahiti dili." (*People at anandwan told me about it.)

Patient 11: *"Doctoranni ithe mala tyachhi salah dila ani shastrakriye purvi sandhyachi takad vadhavnya sathi physiotherapy che vyayam karayla lavle"* (The doctor here advised me about it and made me do the physiotherapy exercises before the surgery to strengthen the muscles).

Recovery Period Post Surgery

The typical time for recovery post-reconstructive surgery of leprosy can vary depending on factors such as the extent of the surgery, individual healing abilities, and the presence of any complications. However, it may generally take several weeks to months for the initial recovery, and then additional time for rehabilitation and restoration of function. Here, participants were asked about their recovery period.

Patient 1: *"Plaster 21 din ke baad nikal dete hai. Uske baad exercises sikhate hai. Operation ke baad koi bhi complication ho, toh wo iss hospital main uska ilaaj karte hai”* (Plaster is cut after 21 days. After removal of plaster, exercises are taught. Post-OP complications were treated at the hospital).

Patient 3 - *“Kahi loka rugnalay sodun nighun ghari jatata" (*Few people leave hospital and go back to home).

Patient 8 - *“Jo ghar nai jaa pata, woh idhr rehte hai kondhwa main”* (People who can’t go back home, stay here in Kondhwa).

Patient 12 - *“Koṇḍhavyamadhe oparesananantara 4-5 mahinansaṭhi physiotherapy che vyayama kele jatata. Oparesananantara majha dainandin kamasthi jaṇyasaṭhi 6 te 7 mahine lagale”* (Physiotherapy exercises are done for 4-5 months post the surgery at Kondhwa. It took me six to seven months to go back to my normal routine after surgery).

Impact of RCS on Patient's Life 

Reconstructive surgery for leprosy patients has a transformative impact, restoring lost functionality and mobility while improving psychological well-being. By correcting deformities and preventing further disabilities, it enhances patients' ability to engage in daily activities and integrate into society. Beyond physical improvements, surgery also addresses social stigma, fostering greater acceptance and participation in community life. Here participants were asked about the impact of RCS on their lives.

Patient 1 - *“Mujhe lagta hai ki iss samaj ne mujhe wapas apna lia hai”* (I felt accepted in the society again).

Patient 3 - “*Mi majhya karyasathi itaravar avlambun naste. Ata mi swatah anna khau shakte”* (I don’t depend on others for my work. I can eat food on my own now).

Patient 4 - *“Maine ab kaam phir se shuroo kar diya hai. Mainne kadhaee mein bhee vistrt kaam karana shuroo kar diya hai” (*I have resumed work now. I have started to do detailed work also in embroidery).

Patient 10 - *“Shastrakriyennantar haat saral jhala, tyanantar mi kaam karu shaklo” (*Hand was straightened after surgery, after which I could work).

Patient 13 - “*Shastrakriyennantar ek company madhe join jhalo*” (I have joined in a company post-surgery).

Change in Attitude of People Around You After the Surgery

Patients who have undergone reconstructive surgery (RCS) experience an improved quality of life compared to their past experiences before the surgery and to those who still hide their deformities and disabilities due to social stigma. This boost in confidence helps them regain acceptance in society. Here the participants were asked about the change in attitude of people towards them once the RCS was done.

Patient 5 - “*Mala adhik atma-vishwas vatla. Mi dukkha rahnyache thambawale aahe*” (I felt more confident. I have stopped feeling sad).

Patient 7 - *“Mala samajat punha svikaar vatla. Tichyachya samajik interaction madhe khup sudharna disali*” (I felt accepted back to the society. I have noticed a dramatic improvement in her social interactions).

Patient 10 - “*Shastrakriyenpurvi, mi bahiskrut vatayche. Ata, mi rastya var chaloo shakto. koni mala baghtay asa watat nahi. Mala jivana madhe dusri sandhi milali asa vatalay”* (Before the surgery, I felt like an outcast. Now, I can walk down the street without feeling everyone is staring at me. I feel like I have a second chance at life).

Patient 11 - “*Kutumb ani samajik sambandh sudharle aahet*” (Family and social relationships have improved).

Incentive Received on Time by You After the RCS

NGO institutions that conduct reconstructive surgery often report that leprosy-affected individuals with disabilities, who are typically poor, are reluctant to undergo surgery despite it being provided free of charge. This reluctance is due to the long hospital stays required, and the economic burden on family members who need to accompany and stay with the patients. To address these challenges, an incentive of Rupees 8000/- which is now increased to 12,000/-is provided to leprosy-affected individuals by the Government of India via National Leprosy Eradication Programme (NLEP) (12). Here the participants were asked about receiving the incentive.

Patient 1: “*20 saal pehle kuch maddat nahi tha Sarkar se. NGO ke log hamara dekh bhal karte the*” (There was no help 20 years ago. NGO looked after us).

Patient 7: “*Mala ek operation sathi 5000 milale. Maze 4 vela operation jhale. Mala velela 20,000 milale*” (I have received 5,000 for one operation. I have been operated on four times. I have received 20,000 on time).

Patient 9 - “*Mala 8000 milale. Ani majha aahar hospital madhe dilela hota”* (I have received 8000. and my food was given in the hospital).

Patient 10 - “*Sarkar kam karaycha agodar disability scheme chalavla jata hota. Telco colony madhil khup lokani hya scheme suru karayla kaam kele. Sarkari scheme antargat, aplyala disabled person mhanun privileges miltaat. PMT madhye free travelling. Mala sarkar kadun ration suddha milala*” (Disability scheme functioned earlier before the government worked. A lot of people in the telco colony worked to start the scheme here. Under the government scheme, we get privileges as a disabled person. Free travelling in PMT. I even got ration from the government).

Patient 11 - “*Majhya grampanchayat ne mala disability schemes madhe yenyas madat keli*” (My grampanchayat helped me get into disability schemes).

Patient 12 - “*Majhi surgery zali aahe. Mala purn vishwas aahe ki mala lavkarach paise miltil”* (My surgery is done. I am fully assured I will get the money soon).

Advice to Give Someone Considering RCS

Here the participants were asked about the advice they would give other leprosy patients about undergoing reconstructive surgeries.

Patient 1 - “*Main surgery ke baad idhr ruk gaya. Aur abhi baaki sabko maddat karta hu*” (I have stayed back here after surgery and now I help around).

Patient 4 - “*Main unko raasta dikhaunga aur bataunga kaise surgery ho sakta hai. Unko zindagi main doosra mauka mile, iska koshish karunga”* (I will guide them to what to do and how things are to be done and where they can find help. I will make sure that people get a second chance in life).

Patient 10 - “*Mi itar lokanna surgery karayla madat ani margadarshan karin*” (I will help and guide other people to do the surgery).

Leprosy patients' qualitative feedback through in-depth interview

**Figure 2:**
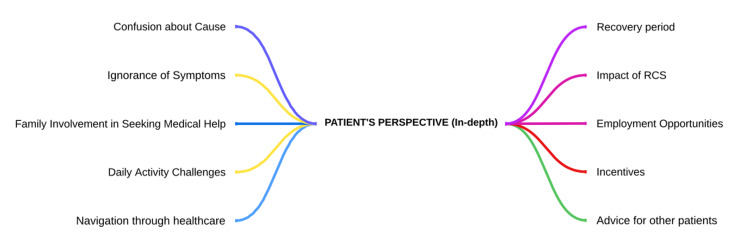
Themes generated from in-depth interviews of the patients

Qualitative feedback was collected from the leprosy patients who underwent reconstructive surgery (RCS) through in-depth interview. Two in-depth interviews were conducted at the rehabilitation centre. Patients who underwent reconstructive surgery for eye operation and ulcers treatment revealed their experiences, emotions, and practical considerations, highlighting the pursuit of treatment, recovery period, life impact, social reactions, incentives, and advice for future patients. These insights underscore the complex journey and multifaceted factors influencing their surgical experiences and the themes generated are depicted in Figure 2.

Confusion About Cause 

The theme "Confusion about Cause" captures the patient's uncertainty and lack of understanding about how they contacted leprosy. This confusion can be rooted in several factors such as limited access to medical information, social stigma, and misconceptions about the disease. Here the participants were asked about their perspective about the disease.

Patient 1 - *“Mala kadhi samajla nahi ki mala ha rog kasa zala. Mi ghabaralo hoto”* (I never understood why I got this disease. I was scared).

Patient 2 - *“Mazha shejari mala sangitla ki kustha rog mazya magachya paapamule ahe” (*My neighbor told me leprosy is because of my past sins).

Ignorance of Symptoms 

The burden of leprosy, particularly new cases with deformities, has been increasing in recent years, presenting a significant public health issue. However, there have been reports of long delays in diagnosis. The delays on the part of patients were longer than those by healthcare providers. Many patients and their family members were unaware of the early symptoms of leprosy. Additionally, limited community activities and misdiagnosis seem to contribute to the ignorance of symptoms. Here participants were asked about knowledge of symptoms

Patient 1 - “*Mala majhya haatawar kahi lal dhaage disle. Mi tyakade laksh dila nahi. Nantar mala kamzori pan janawali”* (I noticed some red spots on my arms. I didn’t pay attention to it. Then later on, I developed weakness).

Patient 2 - “*Mi majhya haatawar ek pandhra daag pahila. Mi majhya mulala sangitla. Tyala vatla nahi ki ha leprosy ahe*” (I saw one white patch on my arms. I told that to my son. He didn't think it was leprosy).

Family Involvement in Seeking Medical Help

The active participation of family members in mitigating the impact of leprosy is crucial for individuals affected by the disease. This support is especially significant in communities where individuals face various clinical, psychological, social, and behavioral challenges due to leprosy.

Patient 2 - “*Maza mulga mala Deenanath Mangeshkar Hospital la gela. Mi ek varsha bhar aushadh ghetle*” (My son took me to Deenanath Mangeshkar Hospital. I took medicines for a year).

Daily Activities Challenge

The deformities from leprosy pose a significant problem in one’s daily activities. The prospect of being able to participate more fully in daily activities can serve as a powerful motivating factor for individuals considering reconstructive surgery following leprosy, helping them regain confidence, independence, and a sense of normalcy in their lives. Here, the participants were asked about the challenges they faced in doing their daily activities.

Patient 1: *"Majhe ole paṇyane bharat hote, Majhe dṛṣṭikṣamata vaḍhat hoti, Mi majhe oḷe micakavaṇyasa asamartha hote*” (My eyes kept watering. My vision was getting bad. I was unable to blink).

Patient 2: *"Majhya hatache bot prabhavit zhale hote. Mala saḍi nesata yeta navhati. Majhya mulavara avalambuna rahave lagale*” My fingers were affected. I could not wear a saree. I had to depend upon my son).

Navigation Through Healthcare

To achieve the goal of eliminating leprosy as a public health issue in India, it is crucial that patients make extensive use of leprosy services provided by the government. Here, the participants were asked about how they got access to the reconstructive surgery process.

Patient 1: “*Mazhya parivaar doctoranni cha sujhav war mazza mevhana mala ithe gheun ala*” (My brother-in-law got me here after my family doctor’s recommendation).

Patient 2 - “*Mazhya doctoranni Deenanath yethe mahiti dili ki mazi shastrakriya hoou shakte asha leprosy hospital la jave*” (Information was given by my doctor at Deenanath to go to leprosy hospital where my surgery can happen).

Recovery Period 

The recovery time following reconstructive surgery for leprosy can differ based on various factors, including the complexity of the surgery, the patient's individual healing capacity, and any complications that may arise. Typically, initial recovery might span several weeks to months, with further time needed for rehabilitation and functional restoration. Participants were asked about their specific recovery periods.

Patient 1 - “*6 saptah lage*” (It took six weeks).

Patient 2 - “*phijiyotherepee kee gaee*” (Physiotherapy was done).

Impact of RCS

Reconstructive surgery for leprosy patients significantly changes lives by restoring functionality and mobility, while also enhancing psychological well-being. Correcting deformities and preventing additional disabilities enables patients to perform daily activities and better integrate into society. Besides physical benefits, the surgery helps reduce social stigma, promoting greater acceptance and involvement in community life. Here participants were asked about the impact of reconstructive surgery on their lives.

Patient 1 - “*Mi changla vatat aahe. Maze dolyan madhey samasya nahi ahe*” (I feel good. My eyes have stopped being a problem).

Patient 2 - “*Farach farak zala aahe. Ata mi kam karu shakto*” (There has been a difference. I am able to do things now).

Employment Opportunities

Reconstructive surgery for leprosy patients profoundly enhances their employment prospects by restoring functionality and mobility, enabling them to perform job tasks they previously could not. The ability to work again brings a sense of purpose and normalcy, significantly enhancing both personal and professional aspects of their lives. Here the participants were asked about their employment outcome post-RCS.

Patient 1 - “*Mala atmavisvasa vaṭato kaam parat karayla. Majha drishti sudharla aahe. Mi bai mhanun kaam karayla suru kela aahe”* (I feel confident to join back to work. I have started to work as a maid).

Patient 2 - “*Mi khupa anandi ahe”(*I feel very happy).

Incentives 

NGO institutions conducting reconstructive surgery frequently observe that individuals affected by leprosy, who often come from impoverished backgrounds, are hesitant to undergo the procedure despite it being offered for free. This hesitation stems from the lengthy hospital stays required and the economic strain on family members who must accompany and stay with the patients. To mitigate these challenges, the Government of India via NLEP is providing an incentive of Rs 8,000/-, now increased to Rs 12,000/-, to those affected by leprosy. Participants were asked about their experiences with receiving this incentive.

Patient 1 - “*Majhi surgery zale aahe. Mala ata paise milnar*” (My surgery is done. I will receive money).

Patient 2 - “*Mala government kadhun paisa milala aahe”* (I have received money from the government).

Advice for Other Patients

Here, the participants were asked were asked about the advice they would give other leprosy patients about undergoing reconstructive surgeries.

Patient 1 - “*Mi yaachi sujhav dete*” (I recommend it).

Patient 2 - “*Mi itar patients la sangin he karayla*” (I will tell other patients to for it).

Qualitative feedback from a surgeon who performs reconstructive surgeries on leprosy patients through in-depth interview

**Figure 3:**
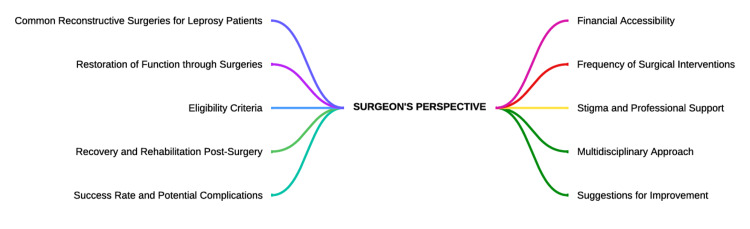
Themes generated from an in-depth interview of the surgeon

One In-depth interview was conducted for a surgeon who works at a tertiary care hospital. Figure 3 depicts briefly the themes and items that were derived from the interview. The verbatims that were quoted directly from the interviewee are written below.

Common Reconstructive Surgeries Performed for Leprosy Patients

“We perform various reconstructive surgeries for leprosy patients, including correction of hand deformities through tendon transfers and claw hand correction. Symbrachydactyly surgery is performed for loss of digits. For foot deformities, we address foot drop and claw toe correction."

"In terms of facial deformities, we treat lagophthalmos with tarsorrhaphy, and for facial nerve palsy, we use dynamic muscle transfers or static slings to restore facial symmetry and function. Additionally, we perform rhinoplasty or nasal reconstruction to correct saddle nose deformities."

"For chronic ulcers, we employ surgical debridement, skin grafting, or flap surgery, especially on the hands and feet. Rehabilitation and functional improvement involve joint stabilization, and in severe cases where reconstruction is not feasible, we may proceed with amputation followed by prosthetic fitting”

Restoration of Function Through Surgeries

"When there is claw hand deformity, the ability to grasp things is lost. Tendon transfers can restore these lost or impaired motor functions, allowing patients to regain the ability to perform daily activities. Correcting foot drop enables patients to walk more naturally without dragging their feet, helping stabilize the foot and prevent further deformities. These surgeries significantly improve the quality of life and the cosmetic appearance of deformed limbs, becoming an integral part of a broader rehabilitation program.”

Eligibility Criteria of Patients for the Reconstructive Surgery

"To be eligible for reconstructive surgery, patients must have completed the scheduled course of MDT, or at least have been on it for six months. They should be free from reactions and symptomatic neuritis for at least six months and should not have had a lepra reaction during the past six months unless the surgery is specifically for neuritis."

"Age as such is not a problem but we prefer not to operate on patients over 70 years of age as recovery isn’t good. The duration of muscle paralysis should be at least 1 year. The suppleness of the joints is a crucial criterion; severe contractures or stiff joints are not suitable for surgery, although physiotherapy or surgery can reverse some contractures. Patients must have no skin infections, deep cracks, wounds, or ulcers at the time of referral. Additionally, there should be no tenderness of any major nerve trunk in the limbs.”

Time Period for Post-operation Recovery and Rehabilitation

“It takes roughly between three to six months for the new normalcy to return.”

Success Rate and Potential Complications

“The success rate of reconstructive surgery in leprosy can be quite high, roughly 85% to 90%, with many patients experiencing significant functional improvements and enhanced quality of life. However, patients with leprosy may experience delayed wound healing. Surgical wounds might reopen or not close properly, leading to complications that require additional interventions. During tendon transfers or other reconstructive procedures, there is a risk of damaging surrounding nerves, which can result in sensory or motor deficits. Additionally, post-operative joint stiffness can occur, particularly if physiotherapy is not adequately followed.”

Financial Accessibility

"Most of the surgeries are performed in government hospitals, where finance doesn’t pose a challenge. Patients who can afford it get operated on in private or semi-private hospitals. As doctors, we mostly try to work around their financial challenges. In fact, most of the surgeries are done pro-bono; we don’t charge at all.”

Frequency of Surgical Interventions Done

“I go to Kondhwa Hospital once or twice a year, depending on the patient load and my availability, to perform surgeries. In private practice, the number of surgeries for Hansen’s I perform is very sparse.”

Stigma and Professional Support

“There is no perceived stigma from other doctors; my fellows are very supportive.”

Multidisciplinary Approach

“Reconstructive surgery for leprosy needs a multidisciplinary team approach to ensure comprehensive care and optimize patient outcomes. Physiotherapists and occupational therapists play vital roles in preoperative assessments, postoperative rehabilitation, and functional improvement. Psychologists provide essential preoperative counseling and ongoing emotional support, addressing body image concerns and promoting mental well-being. Social workers assist with resource coordination, while prosthetists and orthotists design and fit prosthetic and orthotic devices.”

Suggestions 

“There's a need for infrastructure improvement as the hospitals are in a dilapidated condition, and the operating theaters aren't kept in a sanitary state. Additionally, there's a lack of instruments; sometimes, we have to carry our own. Furthermore, there's inadequate staff. So funding needs to be aptly given and on time. Finally, active case detection and treatment by NLEP are crucial to prevent deformities from arising.”

## Discussion

The current study aims to evaluate the quality of life of individuals who have undergone reconstructive surgery (RCS) for leprosy. The qualitative analysis delves deeply into the emotional and social dimensions experienced by leprosy patients undergoing reconstructive surgery (RCS). Participants shared poignant accounts of their initial understanding of leprosy, characterized by fear and despair due to its severe physical manifestations and the resultant social and economic repercussions. Many recounted the distressing loss of jobs and societal exclusion they faced, highlighting the pervasive stigma and discrimination associated with the disease. Despite these challenges, participants expressed a gradual shift in family and social dynamics, ranging from unconditional support to instances of rejection and eviction. This variability underscored the profound influence of familial attitudes on their overall well-being and quality of life.

Regarding RCS, participants described their journey toward seeking surgical intervention as marked by prolonged periods of pain and disability. Their decisions were driven by a strong desire to regain lost functionality and independence, emphasizing the pivotal role of RCS in enhancing their daily lives. However, limited awareness and misconceptions about the surgical procedure were prevalent among participants, contributing to hesitations and fears about hospitalization and anesthesia. The recovery period post-surgery was described as physically and emotionally challenging, varying widely among individuals based on the extent of surgery and access to post-operative care. Despite these challenges, RCS was universally acknowledged for its transformative impact, enabling participants to reclaim mobility, confidence, and a sense of normalcy in their interactions within the community.

Moreover, participants noted positive shifts in societal attitudes following RCS, citing reduced stigma, increased acceptance, and improved social interactions with neighbors and acquaintances. Many expressed gratitude for government incentives that provided crucial financial support during their recovery, highlighting the incentives' role in alleviating economic burdens associated with treatment. Their advice to fellow leprosy patients considering RCS emphasized the importance of timely medical intervention, self-advocacy, and thorough exploration of treatment options, echoing their personal experiences of improved health and well-being post-surgery. Overall, the qualitative study underscores the profound impact of RCS on leprosy patients' lives, advocating for enhanced education, comprehensive support systems, and societal awareness campaigns to foster inclusivity and empowerment.

From the surgeon's viewpoint, reconstructive surgery (RCS) for leprosy patients involves a diverse range of procedures aimed at restoring both function and aesthetics compromised by the disease's debilitating effects. Surgical interventions include tendon transfers for claw hand deformities, corrections for foot drop and claw toes, and treatments for facial nerve palsy and saddle nose deformities. These surgeries are crucial for improving motor function and enhancing the overall quality of life for patients, addressing chronic ulcers through debridement and skin grafting where necessary. Despite the technical success and high satisfaction rates (85-90%), the surgeon highlights significant challenges within the healthcare system that impact patient care.

Infrastructure limitations, including inadequate hospital facilities and shortages of essential surgical instruments, pose substantial obstacles to delivering optimal surgical outcomes. Financial constraints are mitigated in government hospitals where many surgeries are performed pro bono, reflecting the ongoing need for systemic improvements and enhanced funding to sustain these vital services. The recovery and rehabilitation period post-surgery typically spans three to six months, emphasizing the importance of multidisciplinary care involving physiotherapists, psychologists, and social workers to support patients through their healing process.

The surgeon stresses the eligibility criteria for RCS, including completion of multidrug therapy (MDT), absence of recent reactions, and suitable joint conditions. They underscore the critical role of the National Leprosy Eradication Programme (NLEP) in active case detection and treatment to prevent deformities and facilitate timely surgical intervention. Despite these challenges, the surgeon reports strong professional support within the medical community and advocates for ongoing improvements in healthcare infrastructure, resource allocation, and patient education to enhance surgical outcomes and promote comprehensive care for leprosy patients.

In a 2016 study conducted by Debajanee Lenka in Sonepur, Odisha, the entire population of 71 patients who underwent reconstructive surgeries between 2000 and 2012 was considered [14]. Of these, 60 patients were still alive and included in the study, eliminating the need for sampling. Using a semi-structured questionnaire, the study assessed patients' understanding of the surgery and its impact on their quality of life (QOL). The results showed that 98.6% of patients felt their expectations were met. However, only 33.3% of the patients changed their profession post-surgery to mitigate future risks. The study concluded that reconstructive surgery is crucial for reintegrating leprosy patients into normal life and helping them live openly in today's society. These findings closely align with the results of the current study, emphasizing the importance of reconstructive surgery in improving the lives of leprosy patients [14].

Palande and Virmond found that patients who underwent RCS experienced a significant decline in income and acceptance before surgery, which they were able to regain afterward. They emphasized that early correction of disabilities is crucial in preventing further dehabilitation, which is similar to the current study [15].

Ramanathan et al.'s study on 25 patients undergoing corrective surgery found high levels of anxiety and depression preoperatively, with only 40% meeting their expectations post-surgery, contrasting with the present study’s findings. Participants adhered to a six-month rest period post-surgery, following doctors' advice to avoid heavy lifting, which contributed to a high satisfaction rate of 85%, which is similar to the findings of the current study [16].

Recommendations

To effectively combat leprosy and its associated deformities, a multi-faceted approach is essential. This includes enhancing public awareness through campaigns that educate communities, emphasizing early detection, treatment, and the positive outcomes of surgical interventions. Culturally sensitive and multilingual materials should be used to highlight the availability and benefits of reconstructive surgeries. Integrated screening programs must be implemented regularly, especially in endemic areas, targeting high-risk populations such as close contacts of leprosy patients, migrant workers, and residents of slums and rural areas. Service quality in government health facilities needs improvement by ensuring the availability of surgical supplies, drugs, and diagnostic services, along with better reporting systems to boost service utilization.

Accessibility to reconstructive surgeries for leprosy patients should be prioritized, with timely remuneration provided post-surgery. Comprehensive healthcare services should be made available. Specialized training and capacity-building programs for healthcare professionals are crucial, focusing on reconstructive surgeries for leprosy-related deformities. Establishing dedicated reconstructive surgery centers within existing hospitals, equipped with advanced surgical equipment, rehabilitation services, and multidisciplinary support teams, is essential. Community engagement and empowerment should be done to raise awareness, reduce stigma, and encourage early healthcare-seeking behavior. Finally, quality assurance and monitoring mechanisms must be implemented to ensure the safety, efficacy, and ethical standards of reconstructive surgery programs, with regular evaluation frameworks to ensure continuous improvement in service delivery and patient outcomes.

Limitations 

The study relies on convenience sampling, which limits its generalizability due to the non-random selection of participants. The study has been conducted in only one district, hence the findings may not apply to other regions with different social, economic, cultural, and environmental conditions. The relatively small sample size may further restrict the robustness of the results and their broader applicability. The potential for groupthink in focus group discussions may restrict the diversity of perspectives and hinder the exploration of contrasting viewpoints.

## Conclusions

Qualitative findings underscore the profound impact of reconstructive surgery on patients’ lives, demonstrating significant enhancements in functionality, psychological well-being, and social interactions post-surgery. However, challenges such as societal stigma and delays in seeking treatment, often due to fear and a lack of awareness persist. Surgeons highlight the diverse range of reconstructive procedures available for leprosy patients, aimed at correcting deformities of the hands, feet, and face to restore functionality and improve quality of life. In government hospitals, financial barriers are largely mitigated, with many surgeries provided at no cost. The effectiveness of these interventions is further supported by a multidisciplinary approach involving physiotherapists, psychologists, and other specialists. Nonetheless, systemic issues such as inadequate infrastructure, insufficient equipment, and staffing shortages reveal the need for substantial improvements and more proactive case detection efforts by the NLEP.
